# Supplementary material for: Dietary sodium, potassium, and cardiometabolic risk: A cross-sectional analysis of hypertension in U.S. adults from NHANES 2017–2018
Source: PLoS One. 2026 Feb 20;21(2):e0343311. doi: 10.1371/journal.pone.0343311 (PMC12922975; doi:10.1371/journal.pone.0343311)
Supplement: Attachment — Submitted filename: Respones to Reviewers.docx [file pone.0343311.s002.docx]

**Respond to reviewer**

**Reviewer #1**

**Major Comments**

**Comment 1. Definition of Hypertension**

Definition of Hypertension Hypertension is defined solely by self-reported physician diagnosis, which introduces misclassification risk, especially in populations with poor healthcare access. NHANES includes measured BP and guideline-based thresholds (e.g., ≥130/80 mmHg), which should be considered either as primary or sensitivity outcomes to improve validity.

**Response:** We appreciate this important comment. In response, we have clarified the definition of hypertension in the Methods section and acknowledged the potential for misclassification when relying on self-reported physician diagnosis. We also noted that NHANES provides measured blood pressure and medication data that could enable guideline-based definitions (≥130/80 mmHg or current antihypertensive use), which will be considered in future analyses to enhance validity. This limitation has been explicitly discussed in the revised Limitations section.

**Comment 2.Exposure Measurement Bias**

Sodium and potassium intake are estimated from a single 24-hour dietary recall, which is prone to recall bias and daily variability, especially for sodium due to hidden sources. The authors should more clearly acknowledge these limitations and justify the choice not to use urinary biomarkers, which are available in NHANES and are more accurate indicators.

**Response:** We appreciate the reviewer’s thoughtful observation. We agree that estimating sodium and potassium intake from a single 24-hour dietary recall may introduce recall bias and within-person variability, especially for sodium due to hidden sources. In the revised Dietary Assessmentsection, we have explicitly acknowledged these limitations and justified the choice to use dietary recall data rather than urinary biomarkers. Specifically, urinary sodium and potassium measurements in NHANES are available only for limited subsamples, which would substantially reduce sample size and generalizability.

**Comment 3.Null Findings and Statistical Power**

The manuscript reports highly precise null associations (e.g., OR = 1.0000), raising concerns about the exposure scale or measurement error. The authors should assess and report statistical power, particularly for subgroup and interaction analyses.

**Response:** We appreciate this insightful comment. We have clarified in the Results sections that the near-unity odds ratios (e.g., OR ≈ 1.00) are a direct consequence of standardizing all dietary variables (z-scoring) before model entry, meaning that each unit change represents one standard deviation in intake. This approach facilitates comparability but can produce ORs close to 1.00 when effect sizes are small. Regarding statistical power, we acknowledge that although the total sample size was adequate for detecting moderate associations, subgroup and interaction analyses had limited power due to smaller numbers of participants within strata. These clarifications have been added to the revised manuscript.

**Comment 4.Sodium-to-Potassium Ratio**

Although sodium and potassium were analyzed separately, the Na/K ratio, a biologically relevant composite, was omitted. Prior literature supports its stronger association with BP. The authors should include this ratio in their models.

**Response:**

We thank the reviewer for this insightful suggestion. Following the recommendation, we incorporated the sodium-to-potassium (Na/K) ratio into our analysis. As presented in the revised Results section, a higher Na/K ratio showed a positive but non-significant association with hypertension (OR = 1.10, 95% CI: 0.96–1.26, P = 0.18), consistent with the findings for sodium and potassium individually. These additions have been described in the Methods, Results, and Discussion sections.

**Comment 5. Subgroup Analyses and Interpretation**

Subgroup Analyses and Interpretation: While extensive subgroup and interaction analyses are presented, many appear underpowered and lack justification. Interaction p-values should be reported. Claims of “stronger trends” in subgroups without statistical significance should be interpreted cautiously to avoid overstatement.

**Response:** We appreciate the reviewer’s constructive comment. In response, we have clarified that all subgroup analyses were exploratory and that none of the interaction terms reached statistical significance (all P for interaction > 0.05). We have also revised the text in the Results sections to temper the language—changing expressions such as “stronger trends” to “numerically stronger but not statistically significant.”

**Comment 6.Reverse Causality**

The cross-sectional design limits causal inference. Reverse causation is a key concern, individuals with known hypertension may change dietary habits. Stratified models by awareness/treatment status or sensitivity analyses excluding known hypertensives would help address this bias.

**Response:** We thank the reviewer for this important comment. We fully agree that the cross-sectional nature of NHANES limits causal inference and that reverse causation is a potential concern, as individuals with diagnosed hypertension may have altered their dietary sodium and potassium intake. In response, we have revised the Discussion section to explicitly acknowledge this issue and explain that stratified or sensitivity analyses excluding known hypertensive participants would substantially reduce sample size and population representativeness. We have also noted that future longitudinal studies are needed to establish causality.

**Comment 7.Redundancy and Conciseness**

Several results, particularly null associations, are repeated across sections and figures. The manuscript would benefit from a more concise presentation, especially in the Results and Discussion.

**Response:** We appreciate the reviewer’s helpful suggestion. We have carefully revised the Results and Discussion sections to remove redundant statements and streamline the presentation. Repetitive descriptions of null associations have been consolidated, and figure references have been shortened for clarity. These revisions improve readability and conciseness without altering the study’s content or conclusions.

**Minor Comments**

1. Figures and Tables: Ensure all visuals, especially forest plots and splines, are labeled clearly with legends and axes. Some figures (e.g., Fig 4, 5) are described in detail but were not viewable in the review file. Descriptions should match content and be verifiable by readers.

**Response:**

We appreciate the reviewer’s helpful observation. In the revised version, all figures have been reviewed and updated for clarity. Legends now explicitly describe model adjustments, reference categories, and variable units. Axis labels and confidence interval bands have been clearly indicated for the forest plots and restricted cubic spline figures.

2. Terminology: The term “null association” is used frequently. More precise phrasing such as “no statistically significant association” or “no observed association within the intake range” would be preferable.

**Response:**

We thank the reviewer for this valuable suggestion. In the revised manuscript, the term “null association” has been replaced with more precise expressions such as “no statistically significant association” or “no observed association within the intake range,” depending on the context. This change enhances clarity and statistical accuracy without altering the interpretation of our findings.

3. Dietary Fiber Findings: The mention of a possible inverse association with fiber is interesting but underdeveloped. Consider briefly discussing plausible mechanisms or citing supporting studies if retained.

**Response:**
We thank the reviewer for this insightful suggestion. In response, we have expanded the Discussion section to briefly describe plausible mechanisms and supporting evidence for the observed inverse trend between dietary fiber intake and hypertension. Specifically, we note that dietary fiber may influence blood pressure through improvements in endothelial function, sodium excretion, and gut microbiota modulation. Relevant studies from NHANES and meta-analyses have been cited.

4. Ethics Statement: The ethics section is appropriate for secondary analysis of NHANES but contains redundant language regarding consent. Please streamline for clarity.

**Response:**

We appreciate the reviewer’s observation. The Ethics Statement has been streamlined for clarity and conciseness. Redundant language about participant consent and exemption has been removed, while retaining all essential information regarding NCHS ethical approval and public data use. The revised text now reads:

“NHANES protocols were approved by the NCHS Research Ethics Review Board, and all participants provided informed consent. This secondary analysis of de-identified public data was exempt from additional institutional review.”

**Reviewer #2**

**Comment 1.** Why was only one cycle used for this paper when this info is readily available for at least a decade?

**Response:**

We appreciate the reviewer’s observation. Only one NHANES cycle (2017–2018) was used intentionally to ensure internal consistency of variable definitions and dietary assessment methods. Across survey waves, several key updates occurred in nutrient databases, food coding systems, and laboratory calibration procedures, which may limit direct comparability. Additionally, this recent cycle represents the most up-to-date pre-pandemic U.S. population data, allowing the analysis to reflect current dietary and hypertension patterns. We acknowledge that combining multiple cycles could increase sample size, but such an approach would introduce heterogeneity across measurement protocols and weighting schemes.

**Comment 2.** While I appreciate the authors’ effort to demonstrate the robustness of their findings across multiple models, the inclusion of four separate models may be more detailed than necessary. Presenting the unadjusted and fully adjusted models would likely suffice to illustrate the effect of covariate adjustment, while the intermediate models could be summarized in supplementary materials. This would streamline the results and improve interpretability.

**Response:**

We appreciate the reviewer’s thoughtful suggestion. We have streamlined the Results section. The presentation of four models has been condensed into a concise summary sentence describing the stability of the estimates across adjustment levels. This revision improves readability while retaining all essential information. The intermediate models are now summarized descriptively rather than shown in detail, in accordance with the reviewer’s recommendation.

**Comment 3.** The conclusion appropriately reflects the null findings; however, the interpretation could better emphasize the cross-sectional nature of the study. It would be helpful to explicitly acknowledge that causal inferences cannot be made from this design.

**Response:**

We fully agree with the reviewer’s observation. To address this comment, we have revised the Conclusion to explicitly acknowledge the cross-sectional nature of the study and the resulting limitation in causal interpretation. The revised text now reads as follows: “Given the cross-sectional nature of this study, causal inferences cannot be established, and longitudinal investigations are required to confirm these associations.” This addition clarifies that our results describe associations rather than causal effects, thereby improving interpretive accuracy and transparency.

**Comment** 4. the discussion could further explore potential reasons for the lack of significant findings — for example, measurement error in dietary assessment, residual confounding, or limited variability in sodium and potassium intake. This would strengthen the interpretation and provide valuable context for readers.

**Response:**

We appreciate this valuable suggestion. In response, we have expanded the Discussion section to address potential explanations for the absence of statistically significant findings. Specifically, we now note that measurement error in 24-hour dietary recall, residual confounding from unmeasured factors, and limited variability in sodium and potassium intake within the study population could have contributed to attenuated associations.
