# Supplementary material for: Dietary sodium, potassium, and cardiometabolic risk: A cross-sectional analysis of hypertension in U.S. adults from NHANES 2017–2018
Source: PLoS One. 2026 Feb 20;21(2):e0343311. doi: 10.1371/journal.pone.0343311 (PMC12922975; doi:10.1371/journal.pone.0343311)
Supplement: Attachment — Submitted filename: Respones_to_Reviewers_auresp_2.docx [file pone.0343311.s003.docx]

**Respond to reviewer**

**Reviewer #1**

**Comment 1.**

Persisting Concerns About Hypertension Definition The authors acknowledge that hypertension is defined exclusively by self-reported physician diagnosis, but the manuscript still places insufficient weight on the implications of misclassification. - NHANES 2017-2018 contains multiple measured BP readings, yet these data remain unused. The authors note that future analyses "may consider guideline-based thresholds" (Lines 131-135) , but this does not address why these data were not used at least as a sensitivity analysis in the current study. - Self-reported hypertension underestimates prevalence, particularly in younger, uninsured, or low health-care-access groups.

**Response:** We thank the reviewer for this important and detailed comment. We agree that the use of self-reported physician-diagnosed hypertension introduces the potential for outcome misclassification. In the revised manuscript, we have strengthened the Limitations section to provide a methodologically grounded justification for not incorporating guideline-based blood pressure definitions in the current analysis.

Specifically, although NHANES 2017–2018 includes measured blood pressure data, guideline-based classification requires averaging multiple readings and incorporating antihypertensive medication use, which substantially increases missingness and complicates the application of survey weights in fully adjusted models. To maintain analytic feasibility and consistency across covariates, we therefore relied on self-reported hypertension, which provides more complete coverage across sociodemographic groups.

Importantly, we now explicitly discuss the direction of potential bias, noting that underascertainment of hypertension—particularly among younger or lower health–care–access populations—is likely to result in nondifferential misclassification and bias associations toward the null. These clarifications have been added to the Limitations section.

**Comment 2.**

Standardization of Dietary Variables and OR = 1.000 The authors explain that Z-scoring dietary variables leads to odds ratios extremely close to 1.00 (Lines 276-284). While the explanation is correct, the manuscript should still clarify: - Whether the underlying (non-Z-standardized) coefficients show meaningful variation; - What the magnitude of 1 SD of intake represents in mg/day for sodium/potassium. The current presentation risks implying numerical precision that exceeds what the data can supports.

**Response:** We thank the reviewer for this insightful comment. To improve interpretability, we have clarified in the Results section what one standard deviation represents on the original intake scale. Specifically, one SD corresponded to approximately 1,200 mg/day for sodium and 600 mg/day for potassium in this sample. We also note that analyses on the raw intake scale yielded effect estimates in the same direction but with very small magnitudes, consistent with the near-unity odds ratios observed after standardization. This clarification addresses concerns about numerical precision without requiring additional supplementary tables.

**Comment 3.**

Expanded Dietary Variables: Clarify Multicollinearity Risk The multivariable model includes energy, fiber, cholesterol, sodium, potassium, and Na/K ratio together (Lines 313-327). This raises the possibility of: -High collinearity between sodium and energy - Fiber correlating with energy. - Na/K ratio being mathematically related to individual Na and K variables. None of these issues are addressed or tested.

**Response:** We thank the reviewer for raising this important methodological consideration. We agree that several dietary variables included in the fully adjusted models are interrelated and that multicollinearity may be present. Rather than adding additional diagnostics, we have explicitly acknowledged this issue in the manuscript and clarified that collinearity among energy intake, sodium, potassium, fiber, and the sodium-to-potassium ratio may attenuate individual effect estimates and contribute to the observed lack of statistically significant associations. This clarification has been added to the Results section.

**Comment 4.**

Subgroup Analyses—Interpretation Still Overstates Findings The authors softened some language, but several statements still imply meaningful differences where none exist statistically (e.g., “slightly stronger trends,” “more pronounced” in Fig. 6 interpretations). Given the wide confidence intervals and very small effect sizes, these qualitative statements risk overstating findings.

**Response:** We thank the reviewer for this important clarification. In response, we have further tempered the language used to describe subgroup analyses. All subjective descriptors implying meaningful differences (e.g., “slightly stronger trends” or “more pronounced”) have been removed. The revised text consistently emphasizes that, although point estimates differed slightly across subgroups, confidence intervals were wide and overlapping, and there was no evidence of statistically significant interaction. These changes have been applied to both the Results and relevant figure interpretations.

**Comment 5.**

Measurement Error and Reverse Causality While the authors have expanded the Limitations section, some redundancies remain (Lines 534– 545 repeat earlier text nearly verbatim) Moreover: - The potential for dietary modification among hypertensive individuals is acknowledged, but not fully assessed. - Despite their stated concerns about sample size, a sensitivity analysis excluding known hypertensives would still be valuable—even if only descriptive.

**Response:** We thank the reviewer for this constructive comment. First, we have removed the redundant paragraph in the Limitations section (previously Lines 534–545), which repeated earlier discussion of measurement error and reverse causality.

Second, to better contextualize potential reverse causality, we have added descriptive information noting that a substantial proportion of participants with self-reported hypertension also reported dietary sodium restriction, suggesting that post-diagnosis dietary modification may have attenuated observed associations.

Finally, while we agree that a sensitivity analysis excluding known hypertensive participants could be informative, such an approach would substantially reduce sample size and alter population representativeness. Given these considerations, we addressed this issue descriptively rather than through additional exclusion analyses. We believe this approach appropriately balances interpretability and analytic feasibility at this stage.

**Comment 6.**

Inconsistencies Between Abstract and Methods Abstract states sample size = 4,592, but the Results section states 5,569 participants (e.g., Lines 218–219).

**Response:** Thank you for noting this inconsistency. We have corrected the Abstract to reflect the final analytic sample size of 5,569 participants, which is consistent with the Results section. The previously stated value reflected an earlier draft and has now been reconciled. We apologize for the oversight.

**Comment 7.**

Race/Ethnicity Table Appears Incorrect

In Table 1, the racial composition reported for NHANES does not align with population

distribution, and the percentages shown for each hypertension group do not sum to 100% in

several rows.

**Response:**
Thank you for pointing this out. We have carefully reviewed and corrected Table 1. The race/ethnicity distributions and corresponding percentages have been recalculated to ensure internal consistency, and percentages within each hypertension group now sum to 100%. We have also clarified the calculation approach in the table footnote. We apologize for this oversight and appreciate the reviewer’s careful review.

**Comment 8**.

Figures Still Need Additional Clarity Although improved, several figures remain difficult to interpret: - Fig 1 confidence interval bars appear very narrow—authors should clarify resolution and scaling. - Spline plots (Fig 4) lack reference value labeling and units along axes. - Subgroup plots (Fig 5) should explicitly show P-values for interaction.

**Response:** We thank the reviewer for these helpful suggestions. To improve figure clarity, we have revised the figure legends as follows. For Fig 1, we clarified that the narrow confidence intervals reflect near-unity odds ratios and axis scaling rather than unusually high precision. For the spline plots (Fig 4), we now explicitly state the reference intake value and include units for the x-axis. For the subgroup analyses (Fig 5), we have added clarification that no statistically significant interactions were detected (all P for interaction > 0.05). These changes improve interpretability without altering the underlying analyses.

**Comment 9**.

Several typographical issues persist (e.g., duplicated references 8 and 15 appear identical; grammar inconsistencies in Discussion).- Some sections remain overly long; consider additional trimming to improve readability.

**Response:** We thank the reviewer for these helpful suggestions. We have updated the formatting of the references and resolved the issue of duplicate references. We rechecked the entire text and corrected some inconsistent grammatical expressions. Furthermore, we have reduced and refined some of the sections in the discussion. These changes have enhanced readability without altering the original meaning
